# Job satisfaction among people with disabilities in Ethiopia: A cross-sectional survey

**DOI:** 10.4102/ajod.v14i0.1660

**Published:** 2025-07-08

**Authors:** Tsega H. Mirach, Rosemary M. Lysaght, Molalign B. Adugna, Abebe A. Alemu, Meseret H. Ayele, Sewbesew Y. Tilahun, Tewelde G. Adhanom

**Affiliations:** 1 Department of Health Systems and Policy, College of Medicine and Health Sciences, University of Gondar, Gondar, Ethiopia; 2 School of Rehabilitation Therapy, Queen’s University, Kingston, Canada; 3 Department of Sociology, College of Social Sciences and Humanities, The University of Gondar, Gondar, Ethiopia; 4 Faculty of Law, Queen’s University, Kingston, Canada; 5 Faculty of Education, Queen’s University, Kingston, Canada; 6 Department of Psychiatry, College of Medicine and Health Sciences, University of Gondar, Gondar, Ethiopia; 7 Department of Gender and Development Studies, College of Social Sciences and Humanities, University of Gondar, Gondar, Ethiopia

**Keywords:** disability studies, cross-sectional survey, job satisfaction, people with disabilities, higher education graduates, Ethiopia

## Abstract

**Background:**

Job satisfaction among people with disabilities (PWDs) is a significant concern because of its impact on productivity, job retention and well-being in the workplace.

**Objectives:**

This study aimed to assess the job satisfaction of employees with disabilities in Ethiopia and to identify key factors influencing job satisfaction.

**Method:**

A cross-sectional survey was conducted with 784 Ethiopian government employees with various disabilities. All interviews were conducted in 2021.The survey was designed to collect key socio-demographic information, and factors related to job satisfaction.

**Results:**

The majority of respondents had motor difficulties (59%), followed by visual impairments (36.7%). The mean age was 33 years, and 67% were male. Over half of the study participants were first-degree holders, and 80.6% had experienced integrated education. The mean time to secure a job was 15.41 months, with over 18% unemployed for 6–12 months. Job dissatisfaction was influenced by factors such as low salary, gender, service years and lack of personal assistance. Vision impairment correlated with higher dissatisfaction. Overall, around 32.5% reported satisfaction in their job, 44.1% were neutral and 23.4% were dissatisfied. Dissatisfaction rose to 29% when measured using supplementary questions.

**Conclusion:**

The study was the first to examine factors leading to job satisfaction of employees with disabilities in the Ethiopian public sector. Recommendations include social policy adjustments for better working conditions, considering central factors associated with dissatisfaction. The government should explore measures such as employment quotas or wage supplementation to address disparities and ensure reasonable accommodation. Inclusive research methods will assist in leading change.

**Contribution:**

This research contributes nuanced insights into the factors affecting job satisfaction and its complexities among employees with disabilities in the Ethiopian context, emphasising the need for ongoing research to improve worker support structures and inclusive practices in job acquisition and employment.

## Introduction

People with disabilities (PWDs) are individuals with long-term physical, mental, intellectual or sensory disabilities that may hinder their full and effective participation in society (UN 2006). These disabilities can be acquired at birth, through accidents or illness, or as a result of the natural ageing process. According to the World Health Organization (WHO), over 1 billion people are living with some form of disability, making up about 15% of the world’s population. The World Report on Disability estimated that there were 15 million persons with disabilities in Ethiopia, representing 17.6% of the population (WHO 2011). The vast majority of them live in rural areas where access to basic services is limited (Eide & Ingstad 2011).

Employees with disabilities represent an underutilised human resource for organisations. People with disabilities have on average lower levels of employment, job security, income, pay satisfaction, job satisfaction and overall quality of work life, than people without disabilities (World Bank 2019). These disparities exist despite evidence that demonstrates no significant performance and productivity differences between PWDs and those without disabilities (Bonaccio et al. 2020; Lengnick-Hall, Gaunt & Kulkarni 2008).

Recent research shows that job satisfaction among PWDs in Ethiopia is a growing concern (Bitew 2023). Studies have shown that job satisfaction is a predictor of productivity and retention in the workforce (Baart, Schippers & Meta 2019); however, there is evidence that workers with disabilities feel unsupported by their employers, and that this perception is associated with low commitment to the organisation (Bitew 2023). Broader quality of life-focused studies (Abate & Mekonnen 2021; Krupa et al. 2022) have also identified challenges faced by PWDs in employment, identifying a need for more specific research on job satisfaction in this population.

International studies on job satisfaction in employees with disabilities have examined a number of factors related to job satisfaction, generally revealing lower levels of job satisfaction when compared with non-disabled employees. These studies attribute such differences primarily to social factors (e.g. level of job flexibility, social relations with co-workers, workplace discrimination) and pay differentials (Keating et al. 2022; Romeo, Yepes-Baldó & Lins 2020; Schur et al. 2017; Shier, Graham & Jones 2009). Understanding the factors contributing to job satisfaction in this population can inform policies and interventions aimed at improving their employment outcomes and overall well-being and open the door to increased engagement of PWDs in the labour pool (Saleh & Bruyère 2018). There is a lack of published data on job satisfaction among the working population of PWDs in Ethiopia.

The Ethiopian government has enacted and implemented various laws, policies and standards to support PWDs, including their right to productive and decent work (Melles & Korpinen 2009; Sida 2014). However, an estimated 95% of Ethiopians with disabilities live in poverty (Sida 2014). It is important to recognise that PWDs can and want to be productive members of society. Promoting more inclusive societies and employment opportunities for PWDs requires improved access to basic education, vocational training relevant to labour market needs and jobs suited to their skills, interests and abilities, with adaptations as needed (Trezzini et al. 2021). Many societies are also recognising the need to dismantle other barriers – making the physical environment more accessible, providing information in a variety of formats and challenging attitudes and mistaken assumptions related to disability (Bickenbach 2011; Naami 2015; Shaw et al. 2022).

People with disabilities face various challenges in gaining and maintaining employment, including discrimination, access barriers and lack of accommodation (Morwane, Dada & Bornman 2021), which has potential to impact their job satisfaction. This article aims to explore the factors affecting job satisfaction among PWDs in Ethiopia and the coping strategies that policy makers and organisations can adopt to improve it.

## Research methods and design

### Study design and study setting

This study adopted a cross-sectional research design by employing semi-structured questionnaires (Creswell & Creswell 2022). The study covered Oromia, Amhara, Southern Nations and Nationalities People (SNNPR), and Addis Ababa city administration.

### Study population and sampling strategy

The study focused on three disability categories: physical disability, visual disability and hearing impairments, constituting 83.6% of the total population of PWDs in Ethiopia (JICA 2002). The sample included PWD in these categories who were graduates of Higher Education Institution (HEIs), and employed in the civil service. Only those who graduated in the previous 5 years were included to minimise recall bias. The sample size was calculated using the Cochran, Mosteller and Tukey (2006) formula with a 95% confidence interval (CI), ±5% precision level, 50% prevalence, a design effect of 2 and a 5% non-response rate. The target sample size was calculated to be 807 study participants. A multi-stage cluster sampling approach was employed, incorporating probability and non-probability sampling. Simple random sampling was used to sequentially select primary units, starting from higher administrative levels, such as government ministries, and then moving to more localised levels, such as regional bureaus, zonal administrations and districts. From within the selected ministerial districts, systematic random sampling was used to draw the required number of participants proportionate to district size.

### Data collection

This study employed a structured questionnaire to gather data on factors affecting job satisfaction among employees with disabilities, including demographic details, employment characteristics and satisfaction levels (Online Appendix 1). The demographic variables included age, gender, type and onset of disability, presence of personal assistance at home, marital status, educational status and graduation year. Employment characteristics were work history/experience, monthly gross salary and current employer. To assess job satisfaction, the Macdonald and MacIntyre (1997) *Job Satisfaction Scale* was used. This scale uses a Likert-scale to categorise responses into five levels: very dissatisfied, dissatisfied, neither satisfied nor dissatisfied, satisfied and very satisfied.

The questionnaire was translated into Amharic, and 30 trained interviewers conducted the study. Two days of training covered interview conduct, participant rights and ethical considerations.

To enhance the reliability of the study, the questionnaire was pilot-tested over 3 days in areas not included in the actual study. Necessary modifications were made based on the pilot results to ensure clarity and consistency in the questions. In addition, internal consistency of the questionnaire was assessed using Cronbach’s alpha to measure the reliability of the scales used. Cronbach’s alpha to job satisfaction scale was found to be 0.805. Data were electronically entered into KOBO software by the interviewers. All interviews were conducted during 2021.

### Data analysis

Quantitative data were cleaned using standard approaches within the KOBO program. Descriptive statistics and inferential analysis were employed, including ordinal logistic regression using Stata 14. Job Satisfaction Scales were interpreted as: 42–50/very satisfied; 39–41/satisfied; 32–38/neither satisfied nor dissatisfied; 27–31/dissatisfied and 10–26/very dissatisfied according to the recommended scoring procedure.

Ordinal logistic regression analysis assessed associations between independent variables and the dependent variable. The Stata Brant was used to test the overall model’s proportional odds (PO) assumptions and each predictor. The generalised ordered logit model (gologit2) with partial PO was employed to allow for greater model flexibility by relaxing the PO assumption. Analyses were conducted using the gologit2 command in Stata.

In this study, satisfaction was measured on a five-level ordinal scale, requiring the use of four logit models. The logit effects of each variable were therefore determined across four models. Within each model the probability of being in a higher category (‘satisfied’) as opposed to a lower (‘dissatisfied’) category was determined. The comparisons of satisfaction level for each set of categories were calculated as follows: Model 1 contrasts satisfaction levels 2, 3, 4 and 5 against level 1. Model 2 compares levels 3, 4 and 5 with levels 1 and 2. In Model 3, satisfaction levels 4 and 5 were analysed in relation to levels 1, 2 and 3. Finally, Model 4 focused on satisfaction level 5 and compared it to levels 1 through 4. Hence, odds ratios (OR) greater than 1 indicate that higher values of the explanatory variable increase the likelihood of the respondent being at a high satisfaction level, whereas OR less than 1 indicate that higher values of the explanatory variable decrease the likelihood of the respondent being at a high satisfaction level. Depending on the comparison step, how the effect size of the explanatory variable changed could be confirmed. The level of significance, or α, was set at 0.05 for all statistical tests.

### Ethical considerations

Ethical clearance was obtained from the University of Gondar Institutional Review Board (V/P/RCS/2248/2020) and (GRHBS-138-20 TRAQ36030188). Verbal consent was obtained from each participant, and sign language interpretation was used to communicate with participants with hearing disabilities. Data collection ensured privacy and confidentiality, utilising secure cloud-based storage for quantitative data and a virtual secure folder for data.

## Results

### Socio-demographic characteristics of respondents

The sample comprised a mix of backgrounds, with 44% having been raised in urban areas, almost 37% in rural areas and almost one-fifth reporting they had been raised in both settings. Ages ranged from 21 to 62 with a median age of 33 (standard deviation of 8.3 years). The sample was dominated by male respondents, who comprised close to 70% of the sample. Most were married and 41% of participants lived with their spouse and children. Almost 20% lived alone. Fewer than half of respondents were receiving personal assistance at home. Almost one-third of the sample received government financial assistance.

### Types and levels of disabilities

While the sample included people with motor, visual and hearing impairments, the greatest proportion reported motor impairment (59.2%) as their primary source of disability, and 30% did not require mobility aids. About 26% of the sample reported the use of mobility aids (such as braces, prosthetic devices and crutches), and just 4% were wheelchair users. The next most prevalent group was those with visual impairments (close to 40% of the sample) and 27% reported total blindness. Only about 4% of the sample had hearing impairment, with half reporting total deafness. Just 0.6% of study participants reported more than one type of impairment (see Table 1).

**TABLE 1:** Types and level of disability as reported by participants.

Types and level of disability	Frequency (*n*)	(%)
**Visual impairment**	288	36.7
Total blindness	211	26.9
Partially sighted without visual aids	40	5.1
Partially sighted with visual aids	37	4.7
**Hearing impairment**	33	4.1
Profoundly deaf	17	2.2
Hard of hearing without hearing aids	11	1.4
Hard of hearing with hearing aids	5	0.6
**Motor difficulty**	464	59.2
Physical disability, using wheelchair for most or all mobility	28	3.6
Physical disability, requiring aids for independent mobility (e.g. brace, prosthetic device, crutch)	204	26.0
Physical disability, not requiring mobility aids	237	30.2
**More than one impairment**	5	0.6
Visual impairment and motor difficulty	4	0.5
Visual impairment, hearing impairment and motor difficulty	1	0.1

Note: Some respondents chose more than one category.

Just 11% of the reported impairments were congenital, with most having acquired a disability after birth. Most sources of disease or illness resulting in disability were ‘unspecified’. Most accidents had occurred in the home or residential area (23.6%), but other sources included war or conflict and motor vehicle accidents. A total of 23% were unaware of the source of their impairment.

### History and current educational status of participants

Most participants, 70.5% from first cycle and 80.6% from the second cycle of education, had received their basic education in integrated/inclusive schools. Only 17.3% in the first cycle and 10.6% in the second cycle received their education in boarding schools. Few participants, even 9.8% in the first cycle and 5.9% in the second cycle had attended special schools or classes. For a small number of participants, 2.3% and 2.9%, the impairments were sustained after first- and second-cycle education had been completed.

In terms of higher education, 25% of the sample had received a diploma, while just over half of these were earned in private institutions. About two-thirds (67.7%) of the sample had obtained first-degrees, and the majority of these had been earned in government-run institutions. Only 6.9% held a master’s degree, with 87% of those having been earned in government institutions.

### Job acquisition, tenure and earnings

Over half of the participants (56.1%) had been unemployed for more than 3 months before securing their current job, with 21.0% unemployed for less than 6 months and 13% for more than 18 months. The average unemployment duration was 15.41 months (95% CI: 13.57–17.25). While 58.5% of employees stayed at their first job, 8.8% had worked at over three organisations. Key reasons for leaving previous jobs included salary dissatisfaction (4.7%), discrimination (2.0%) and inadequate disability accommodation (2.0%). Median work experience was 5.0–6.5 years; 33.2% had less than 3 years, and 24.1% had over 9 years. Most employees (59.3%) worked at district or woreda-level organisations, with a median monthly salary of 6073 Birr (220.8 USD); salaries ranged from 1 585 Birr (57.6 USD) to 76 625 Birr (2786.4 USD).

### Job satisfaction of respondents

**FIGURE 1:**
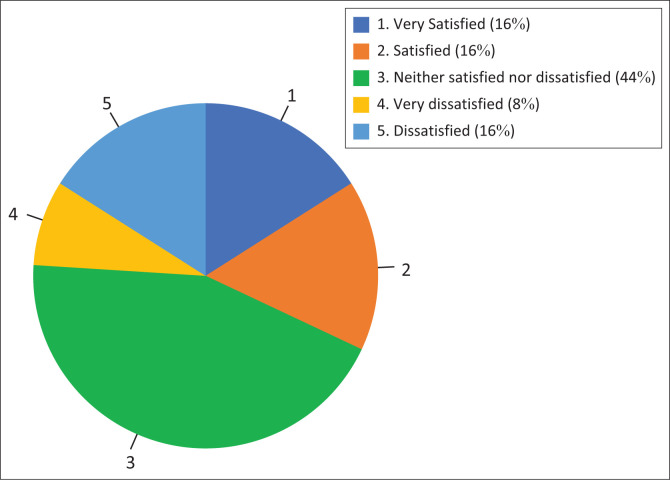
Reported job satisfaction level of respondents.

Respondents were asked to choose from a Likert-scale (Very Satisfied to Very Dissatisfied) their level of satisfaction with their current employment. Overall, 32.5% indicated that they were satisfied or very satisfied, and another 44.1% selected the middle of the scale, leaving 23.4% dissatisfied or very dissatisfied (Figure 1).

### Generalised ordinal logistic regression of predictors to job satisfaction levels

A partially constrained generalised ordinal logistic model was fitted to estimate the ordinal outcome variable ‘job satisfaction level’ from a set of predictor variables that included age, gender, types of impairment, first occurrence of the impairment and presence of personal assistance at home, monthly gross salary, marital status, working experience, graduation year, educational status and current employers. The results of this analysis are shown in the Tables further in the text, organised by level of job satisfaction. Statistically significant findings by category are discussed further in the text.

### Effect of predictors on ‘very dissatisfied’ job satisfaction levels

For employees who reported being very dissatisfied with their work, the odds of being in a higher satisfaction category were 5.04 times greater (95% CI: 1.51–16.76) for those earning more than 8000 ETB compared to those earning less than 4000 ETB (see Table 2).

**TABLE 2:** Generalised ordinal logistic regression outputs showing the effect of predictors on job satisfaction level.

Variables	Job satisfaction level							
	Very dissatisfied		Dissatisfied		Neither satisfied nor dissatisfied		Satisfied	
	OR	95% CI	OR	95% CI	OR	95% CI	OR	95% CI
**Age categories (in years)**								
21–28	-	1	-	1	-	1	-	1
28–33	0.99	0.44, 2.22	1.04	0.65, 1.68	0.72	0.38, 1.39	1.74	0.33, 9.26
33–40	0.78	0.32, 1.91	1.10	0.64, 1.90	1.21	0.61, 2.43	3.47	0.83, 14.57
40–62	0.56	0.20, 1.55	0.98	0.53, 1.82	1.59	0.74, 3.42	3.18	0.46, 21.25
**Gender**								
Male	-	1	-	1	-	1	-	1
Female	1.14	0.61, 2.14	1.12	0.78, 1.62	0.68	0.42, 1.10	0.24*	0.07, 0.79
**Type of disability**								
Visual impairment	-	1	-	1	-	1	-	1
Hearing impairment	1.91	0.38, 9.54	1.52	0.67, 3.46	1.57	0.48, 5.13	0	0, 0
Motor difficulty	1.42	0.75, 2.69	2.07*	1.43, 2.99	2.48***	1.59, 3.90	5.81**	2.06, 6.37
More than one impairment	0.14	0.02, 1.07	0.18	0.02, 2.01	0	0.00, 0		1 Omitted
**Onset of disability**								
Acquired after birth	-	1	-	1	-	1	-	1
Congenital from birth	1.23	0.49, 3.09	1.07	0.63, 1.82	1.32	0.71, 2.47	0.50	0.12, 1.99
**Presence of personal assistance at home**								
Yes	-	1	-	1	-	1	-	1
No	0.96	0.53, 1.73	0.80	0.56, 1.15	0.53**	0.35, 0.81	0.37*	0.17, 0.85
**Monthly gross salary (in ETB)**								
< 4000	-	1	-	1	-	1	-	1
4000–6000	1.14	0.55, 2.39	1.82*	1.12, 2.97	1.50	0.77, 3.25	2.99	0.76, 11.72
6000–8000	1.29	0.55, 3.00	1.59	0.94, 2.73	1.06	0.50, 3.51	0.85	0.22, 3.40
≥ 8000	5.04**	1.51, 6.76	11***	2.17, 7.75	2.01	0.95, 6.52	1.45	0.35, 5.93
**Current marital status**								
Married	-	1	-	1	-	1	-	1
Single	0.88	0.45, 1.74	1.24	0.84, 1.84	1.12	0.69, 1.79	1.79	0.69, 4.65
Other	0.35	0.12, 1.14	0.55	0.23, 1.29	0.43	0.12, 1.59	0.57	0.03, 0.68
**Educational status**								
Diploma	-	1	-	1	-	1	-	1
First degree	0.98	0.50, 1.95	1.13	0.73, 1.75	1.93*	1.04, 3.58	1.53	0.44, 5.27
Master degree	0.67	0.16, 2.77	1.32	0.53, 3.27	2.29	0.91, 5.76	2.87	0.49, 6.89
**Graduation year**								
< 2000	-	1	-	1	-	1	-	1
2000–2005	0.55	0.18, 1.70	0.40**	0.21, 0.79	0.35**	0.18, 0.68	1.33	0.37, 4.79
2005–2010	0.53	0.17, 1.69	0.63	0.32, 1.25	0.61	0.32, 1.15	1.48	0.39, 5.65
2010–2013	0.57	0.16, 2.11	0.51	0.24, 1.11	0.40*	0.18, 0.90	0.58	0.12, 2.80
**Work experience (in years)**								
< 3	-	1	-	1	-	1	-	1
3 to 5	1.29	0.60, 2.75	1.17	0.72, 1.88	0.82	0.45, 1.50	1.43	0.44, 4.68
5 to 9	1.08	0.49, 2.37	0.96	0.59, 1.56	0.91	0.50, 1.65	0.87	0.24, 3.23
≥ 9	1.17	0.49, 2.80	0.93	0.54, 1.59	0.55	0.29, 1.06	0.18*	0.05, 0.67
**Current employer**								
District (woreda-level) employee	-	1	-	1	-	1	-	1
Federal government	0.86	0.09, 8.35	1.07	0.24, 4.84	4.78*	1.13, 0.30	0.81	0.07, 9.22
Regional/Addis-Ababa city administration	1.2	0.63, 2.28	1.46*	1.00, 2.14	2.10**	1.36, 3.23	0.77	0.33, 1.81
Zonal government	0.54	0.21, 1.37	1.05	0.54, 2.05	1.36	0.64, 2.89	0.39	0.07, 2.15

OR, odds ratios; CI, confidence interval; ETB, Ethiopian Birr.

* *P* ≤ 0.05;

** *P* < 0.01;

*** *P* < 0.001.

While not technically reaching the level of significance, respondents with more than one impairment were much less likely than those with vision impairment or any other single impairment (hearing, motor) to be satisfied in their work. No other variable type produced odds differences at significant levels.

### Effect of predictors on ‘dissatisfied’ job satisfaction levels

In this second model, salary was also a significant determinant of higher level job satisfaction. The odds of being in the higher order category of satisfaction level for employees who were paid between 4000 and 6000 and more than 8000 ETB were 1.82 (95% CI: 1.11–2.96) and 4.11 (95% CI: 2.17–7.77) times higher than those who were paid less than 4000 ETB, respectively (see Table 2).

The odds of being in the higher order category of satisfaction level for people having motor difficulties were 2.07 (95% CI: 1.43–2.99) times higher than for people with visual impairment. The odds of being in the higher order category of satisfaction level for people who graduated between 2000 and 2005 were 0.40 (95% CI: 0.21–0.79) times less than for those who graduated before 2000.

### The effect of predictors on ‘neither satisfied nor dissatisfied’ job satisfaction levels

In this category, gender, type of impairment, availability of personal assistance at home, education level, date of graduation, level of government office employees were employed in and salary were all significant determinants of satisfaction. The odds of being in the higher order category of satisfaction level for females were 0.66 (95% CI: 0.46–0.94) times *less* likely than for males when keeping the other variables constant. The odds of being in the higher order category of satisfaction level for people having motor difficulties were 2.48 (95% CI: 1.59–3.90) times *higher* than for people with visual impairment when keeping the other variables constant. The odds of people that did not have personal assistance at home were 0.53 (95% CI: 0.35–0.80) times less likely than their counterparts (see Table 2).

The odds of people having a first degree were 1.93 (95% CI: 1.04–3.58) times higher than diploma holders. The odds of being in the higher order category of satisfaction level for people graduated between 2000 and 2005 and 2010 to 2013 were 0.35 (95% CI: 0.18–0.68) and 0.40 (95% CI: 0.18–0.90) times less than for those graduated before 2000. The odds of being in the higher order category of satisfaction level for people working in regional/AA city administrations were 2.10 (95% CI: 1.36–3.23) times higher than for those people working at district/woreda levels. People who paid a gross monthly salary of 4000–6000 ETB, 6000–8000 ETB and ≥ 8000 ETB had odds that were 1.98 (95% CI: 1.21–3.25), 2.13 (95% CI: 1.29–3.51) and 3.963 (95% CI: 2.41–6.52) times higher than those who paid less than 4000 ETB.

### Effect of predictors on ‘satisfied’ job satisfaction levels

In this fourth model, the odds of being in the higher order category of satisfaction level were significantly related to sex/gender, disability type, availability of personal assistance at home and number of working years. For females, the odds of higher levels of satisfaction were 0.24 (95% CI: 0.07–0.79) times less likely than for males. The odds of being in a higher category of satisfaction level for individuals with motor difficulties were 5.81 times higher (95% CI: 2.062–16.388) than for those with visual impairments (see Table 2).

The odds of being in the higher order category of satisfaction level for people who did not have personal assistance at home were 0.37 (95% CI: 0.16–0.85) times less likely than their counterparts. The odds of being in the higher order category of satisfaction level for people who have been working more than 9 years were 0.18 (95% CI: 0.05–0.67) times less likely than for those working less than 3 years, respectively.

## Discussion

The types and levels of disabilities observed in the study shed light on the diverse challenges faced by individuals with disabilities related to employment. Employees with motor difficulty dominated the sample, and notably, a substantial proportion of participants with motor difficulty did not require mobility aids, which may suggest that people with less severe impairments are most likely to be hired into public sector jobs. The predominance of males in the sample (almost 70% compared with 30% females) was higher than the proportion of males in the general labour market, where data show that 69% of adult males are employed, compared with 50.2% of females (a factor of 1.4) (Ethiopian Statistical Service [ESS] 2021). The gender breakdown in our sample reflects the proportion of males and females with disabilities who are working in the public sector; this suggests that gender may be a negative factor for hiring of disabled women (AFB 2023). National data support our findings, given that women with disabilities have somewhat higher rates of unemployment compared with men in both urban and rural areas (ESS 2021).

The educational attainment of participants varied, with a significant proportion holding first-degrees. The dominance of government-run educational institutions in providing education to participants, especially for first-degrees, indicates the role of public institutions in shaping the educational landscape for individuals with disabilities, and might serve as a site for intervention.

Various factors can influence employee satisfaction. Interestingly, a significant proportion of participants exhibited ambivalent attitudes towards their job satisfaction, with the majority reporting a neutral stance – neither satisfied nor dissatisfied. This lack of clearly defined attitudes should not be dismissed outright, as there may be disability-related and other social factors contributing to this ambivalence. For example, a Korean study showed that job satisfaction was higher for women than men, likely because their expectations were lower; however, job satisfaction was lower in more highly educated women, a fact the researchers attributed to higher job expectations in this group (Yu & Choe 2021).

Digging deeper, job satisfaction among participants emerged as a complex phenomenon. Overall, a considerable proportion expressed dissatisfaction with their employment, indicating potential challenges in the work environment. The ordinal logistic regression models provided valuable insights into the factors influencing different levels of job satisfaction. Monthly gross salary emerged as a significant predictor across multiple satisfaction levels, with higher salaries associated with greater satisfaction. This finding underscores income level as a crucial determinant of job satisfaction among individuals with disabilities.

The presence of motor difficulties was consistently associated with higher dissatisfaction levels, indicating the unique challenges faced by this subgroup in the workplace. Additionally, the educational status of participants played a role, with lower satisfaction levels observed among those with first-degrees, especially those who graduated between 2000 and 2005. This finding suggests potential disparities in employment opportunities or attention to workplace accommodations for individuals with disabilities based on their educational background.

Overall, about 32.5% of participants expressed satisfaction with their jobs compared to those experiencing dissatisfaction (23.4%). According to a report by the Danish National Centre for Social Research, PWDs in that country are just as satisfied with their working conditions as people without disabilities and tend to express high levels of job satisfaction overall, although they have a lower sense of job security (Hansen & Nielsen 2008). It is encouraging in the Danish study to see that some PWDs are expressing high levels of job satisfaction; however, this finding contradicts other research that highlights a lack of appropriate workplace accommodation for employees with disabilities and associated worker dissatisfaction (Hansen & Nielsen 2008). The discrepancy raises questions about the expected link between the absence of such accommodation and job dissatisfaction. The reasons behind some employees with disabilities reporting satisfaction without accommodations and others remaining dissatisfied despite receiving accommodations are not clear, underscoring the complexity of factors influencing job satisfaction (Pagan & Malo 2021).

However, it is notable that only 16.7% of this sample reported being highly satisfied with their jobs. This contrasts with the 44.3% of employees with disabilities in an American survey who reported being highly satisfied with their jobs (Sundar et al. 2018). The reported dissatisfaction among 24% of our participants is also of note, suggesting that employees with disabilities may experience dissatisfaction for various reasons. Several additional factors were identified as significantly influencing their job satisfaction or dissatisfaction. These factors include the proximity of workplaces, gender, type of impairment and salary. Those working in Addis Ababa and regional offices reported higher satisfaction levels, possibly because of the greater accommodation availability in urban areas. This finding underscores the existing gap in accessibility between rural and urban areas, contrary to the government’s commitment expressed through the ratification of the Convention on the Rights of People with Disabilities (CRPD) (UN, 2006).

Our study found that women employees with vision impairments reported lower satisfaction levels. These findings echo results of a literature review conducted by the American Foundation for the Blind (AFB), where women with disabilities were less likely to receive accommodations and had lower levels of higher education achievement, contributing to reduced success and job satisfaction (AFB 2023). The challenges faced by persons with vision impairment in our sample are also consistent with the findings of a Norwegian study, which found that people with visual impairments had lower employment rates than the general population. However, employed participants had lower levels of depression and higher levels of life satisfaction compared to other non-disabled employees. Brunes and Heir (2022) suggested that employment plays an important role in general well-being, even if job satisfaction is not always high.

Salary emerged as a crucial factor affecting job satisfaction. This resonates with other research, which revealed that a higher salary is critical for employees with disabilities to offset the additional living costs associated with disability (Banks et al. 2022). A study by Baumgärtner et al. (2015) found that employees with disabilities are less satisfied than their colleagues without disabilities in highly centralised environments. The study found that a decentralised organisational context relates to higher job satisfaction levels for all employees, especially for those having a disability.

Overall, it is apparent that measures that have been put in place to ensure equitable opportunities in education and employment fall short of creating meaningful and satisfying jobs for many aspiring employees with disabilities. Past research has demonstrated that social barriers to employment of employees with physical disabilities, including stigma and negative attitudes, are just as important as addressing physical barriers (Padkapayeva et al. 2017) and this is consistent with the current study. Recent studies have also pointed to the need for solutions to occur at multiple levels. It is insufficient to assume that greater government intervention will lead to better outcomes. As Shaw et al. (2022) concluded relative to their qualitative evaluation of one programme developed to enhance employment outcomes in developing countries, including three African countries, policy makers and planners must engage PWDs in ‘deeper problem analysis’ if they wish to advance a broader approach to the issue and create better opportunities for decent and meaningful work across a range of workplaces. There is evidence in the international literature that greater responsiveness to the accommodation needs of employees with disabilities can improve job satisfaction for those employees and aid in retention (Padkapayeva et al. 2017; Varekamp et al. 2011). This may be an important future direction for research and development in the Ethiopian context.

### Strengths and limitations

The study’s strengths include its comprehensive exploration of socio-demographic characteristics, types of disabilities, educational backgrounds and job satisfaction among individuals with disabilities through an interdisciplinary research team. However, certain limitations should be acknowledged, including reliance on self-reported data and the potential for recall bias of the study participants. Another limitation of the study is the exclusion of the Tigray region because of the ongoing conflict. Because the study identified many interesting correlations for which specific predictor variables were not included, additional study of accommodation factors in each of the populations studied and their relationship to job satisfaction would be warranted.

## Conclusion

The present study is the first to systematically examine the job satisfaction of PWD in Ethiopia. The study was conducted in major regions, including the capital city, Addis Ababa. Several implications can be drawn from this study. Around one-third of the respondents reported dissatisfaction with their current employment conditions. Factors such as being male, having a motor impairment, having a high level of education, having a better salary, having personal assistance at home and working for the federal government or regional government and Addis Ababa city administration were significantly associated with better job satisfaction. Hence, the government, which was the employer in this study, needs to consider some social policy adjustments to improve the adverse conditions faced by employees with disabilities, particularly disabled women and persons with visual impairment. This might include a mandatory employment quota system or wage supplementation.

### Implications and recommendations

In this study, monthly gross salary emerged as a crucial predictor, emphasising the importance of compensation as a salient factor to be addressed. A motor difficulty was consistently linked to higher dissatisfaction, necessitating targeted support. Gender disparities, particularly for women with disabilities, require attention in accommodation rates and education. Promoting employees with disabilities to leadership roles could serve as a benefit to both the employees themselves and their workplace. To address dissatisfaction concerns, employers should ensure competitive salaries, targeted support for motor difficulties, and foster a decentralised, inclusive work environment, potentially retaining valuable employees with disabilities. Ethiopian policy makers should work in partnership with persons with disabilities to consider meaningful strategies for improving access to a broad range of meaningful jobs that support employees’ employment goals. Future research could delve deeper into specific workplace accommodations and interventions that contribute to higher job satisfaction among individuals with disabilities.
